# Supplementary material for: Disrupting upstream translation in mRNAs is associated with human disease
Source: Nat Commun. 2021 Mar 9;12:1515. doi: 10.1038/s41467-021-21812-1 (PMC7943595; doi:10.1038/s41467-021-21812-1)
Supplement: Supplementary file 1 — Supplementary Information [file 41467_2021_21812_MOESM1_ESM.pdf]

Supplementary Figures and Tables for Disrupting upstream translation in mRNAs is associated with loss-of-function in human disease

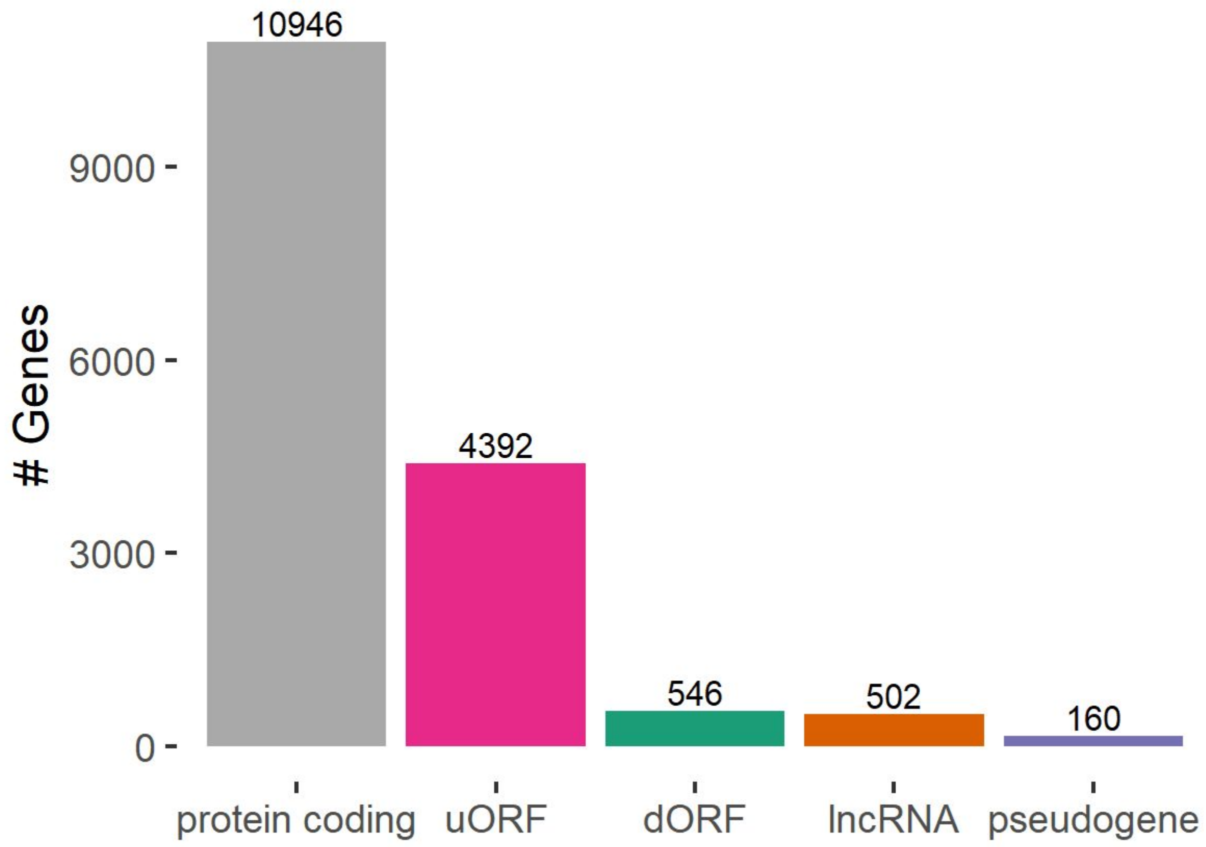

Supplementary Figure 1: Distribution of protein coding ORFs, uORFs, and other non-canonical ORFs mapped by ribosome profiling from Ji et. al paper[16]. dORFs represent ORFs mapped in 3'UTRs, lncRNAs represent ORFs mapped in long-noncoding RNAs, and pseudogenes represent translated pseudogenes respectively.

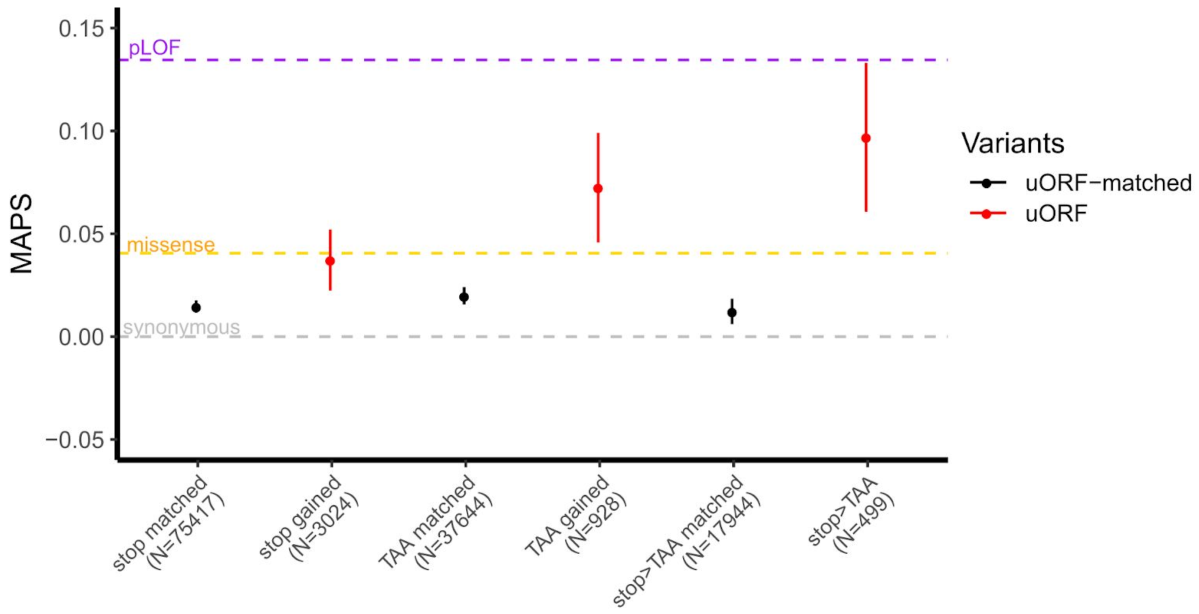

Supplementary Fig. 2: Points showing MAPS scores for uORF UTC-creating and stop-strengthening variants compared to non UTC-creating or stop-strengthening uORF variants matched by trinucleotide mutation context. Error bars represent bootstrapped 90% confidence intervals.

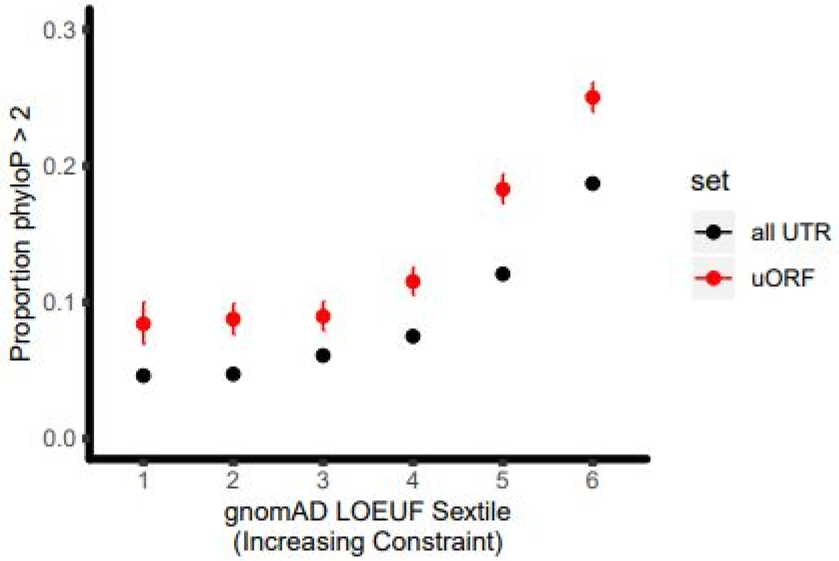

Supplementary Fig. 3: PhyloP scores for possible UTC-creating positions in translated uORFs (red) compared to 5'UTR sequences (black) across all sextiles of gene constraint as determined by gnomAD LOEUF scores (1 being least constrained, 6 being most constrained).

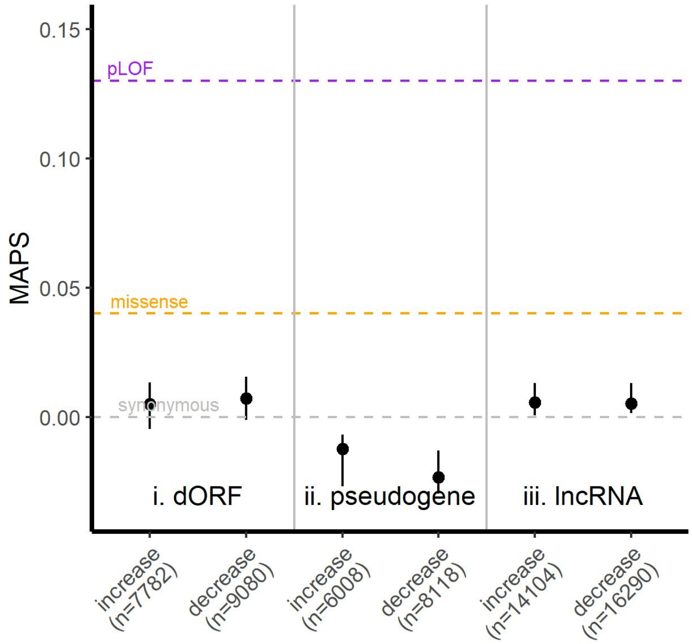

Supplementary Fig. 4: Optimality changing MAPS scores for SNVs in dORFs (3'UTRs), pseudogenes, and long-noncoding RNAs (lncRNAs). Error bars represent bootstrapped 90% confidence intervals.

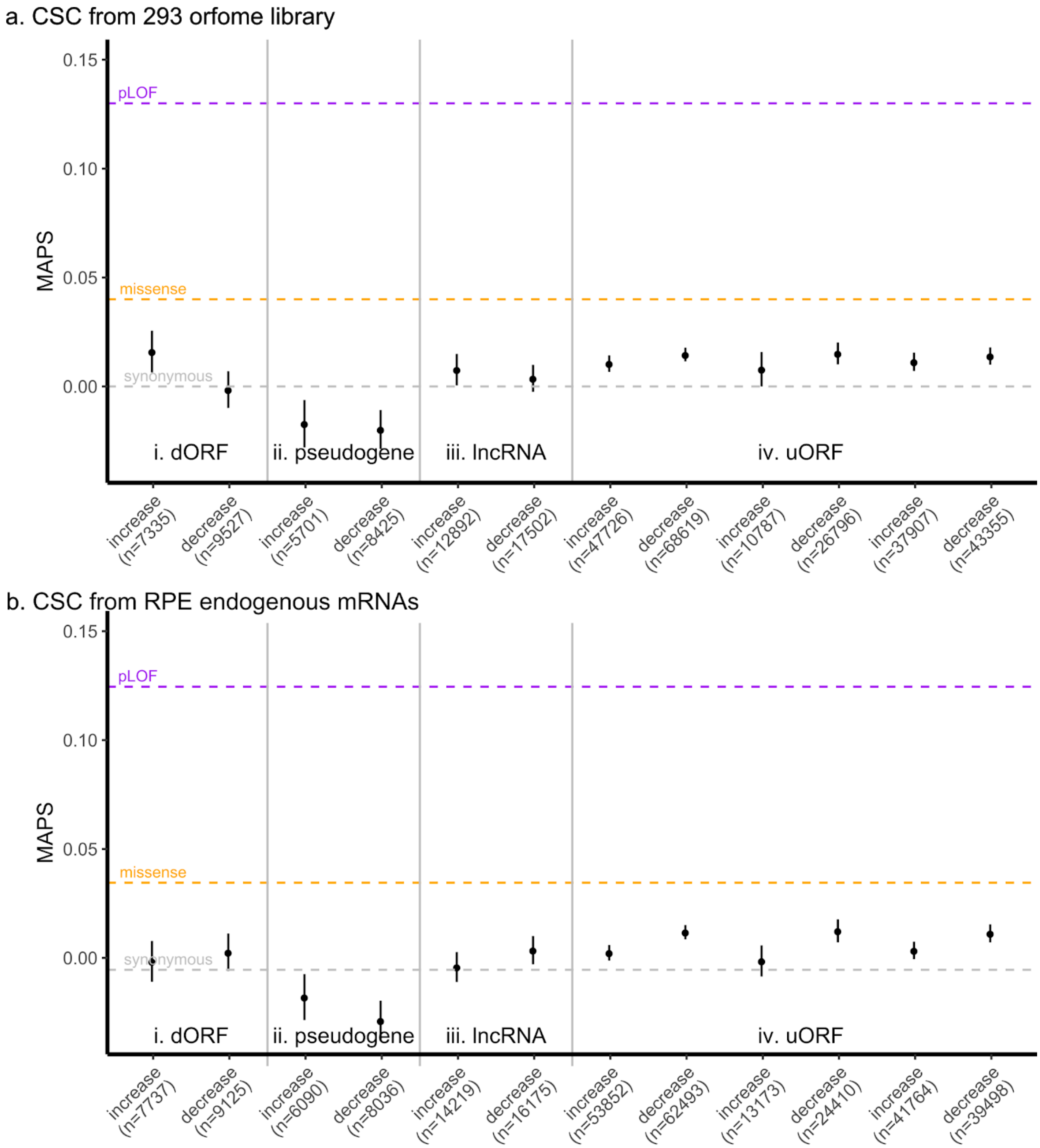

Supplementary Fig. 5: MAPS score dependence on whether a SNV increases or decreases codon optimality in uORFs is robust to changing CSC-scores used to calculate codon optimality across 293 cell lines using the orfome approach, and from retinal pigment epithelium cells with CSC-scores calculated using endogenous mRNAs [35]. Error bars represent bootstrapped 90% confidence intervals.

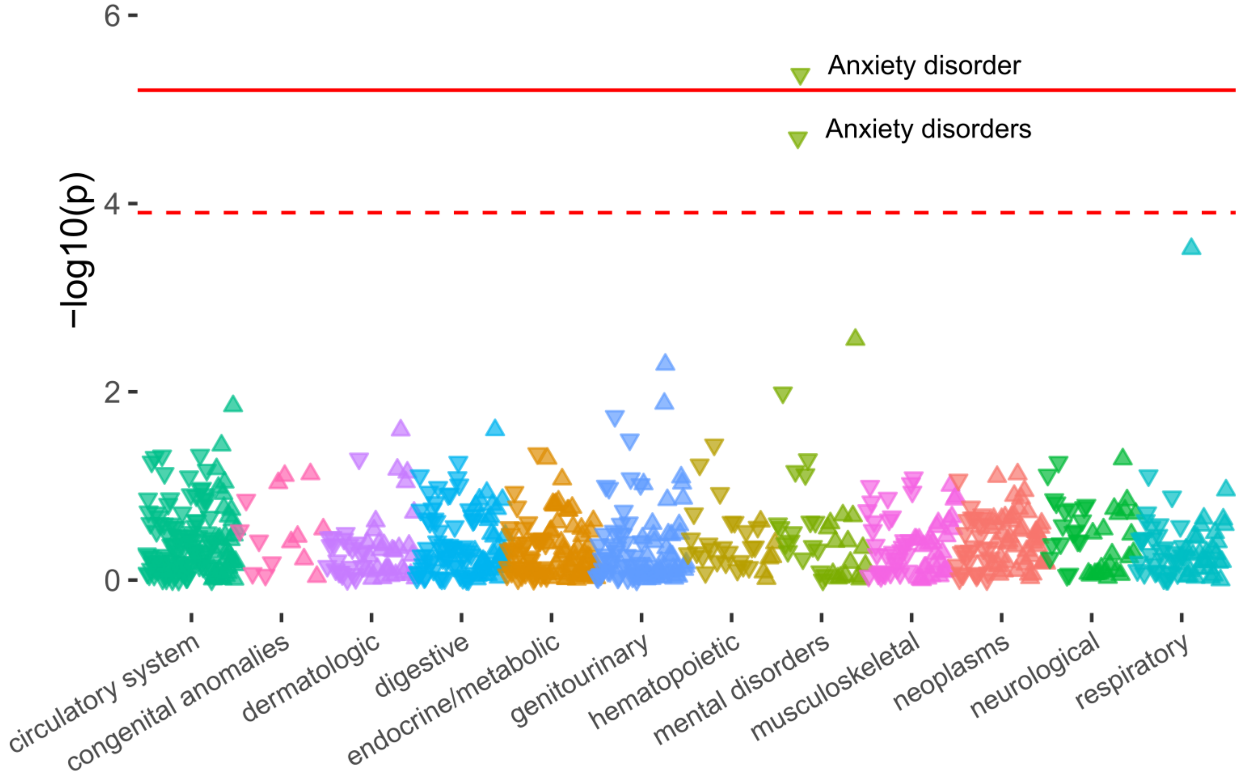

Supplementary Figure 6: PheWAS plot of *VPS53* stop-strengthening variant. The solid red line represents the threshold for Bonferroni-adjusted significance (P=6.25e-6) and the red dashed line represents the FDR threshold (P=1.25e-4) adjusted for multiple hypothesis testing. The direction of each arrowhead corresponds to increased risk (up) or decreased risk (down). P-values are derived from a logistic regression model. Red solid line indicates Bonferroni significance threshold (P=6.25e-05). Red dashed line represents the FDR < 0.1 threshold.

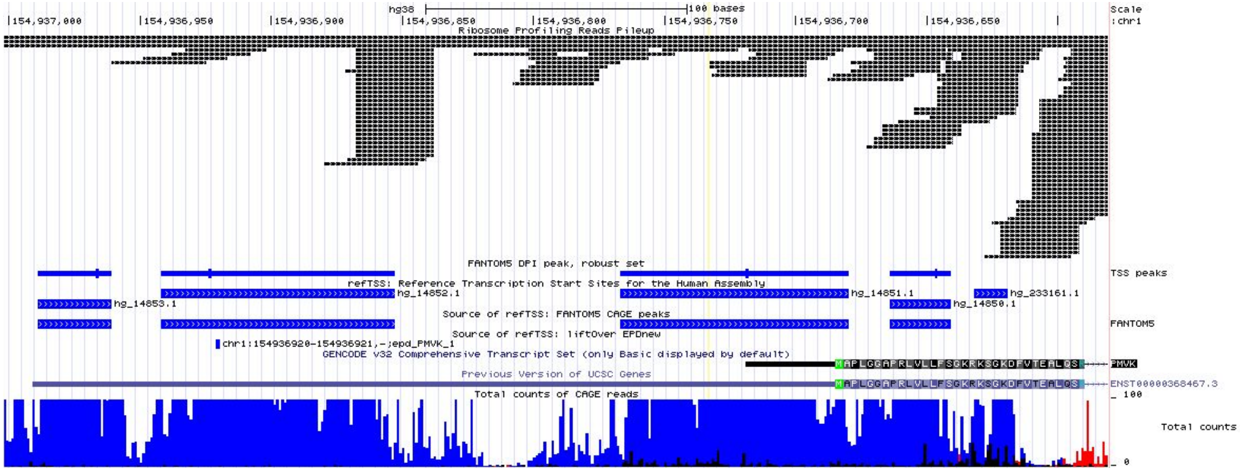

Supplementary Figure 7: Change in PMVK 5'UTR annotation as of September 2019 Gencode 32 release. The longer 5'UTR isoform for PMVK is supported by transcription start-site mapping from FANTOM5, and by the remapped ribosome-profiling reads (top, black) from GSE65885.

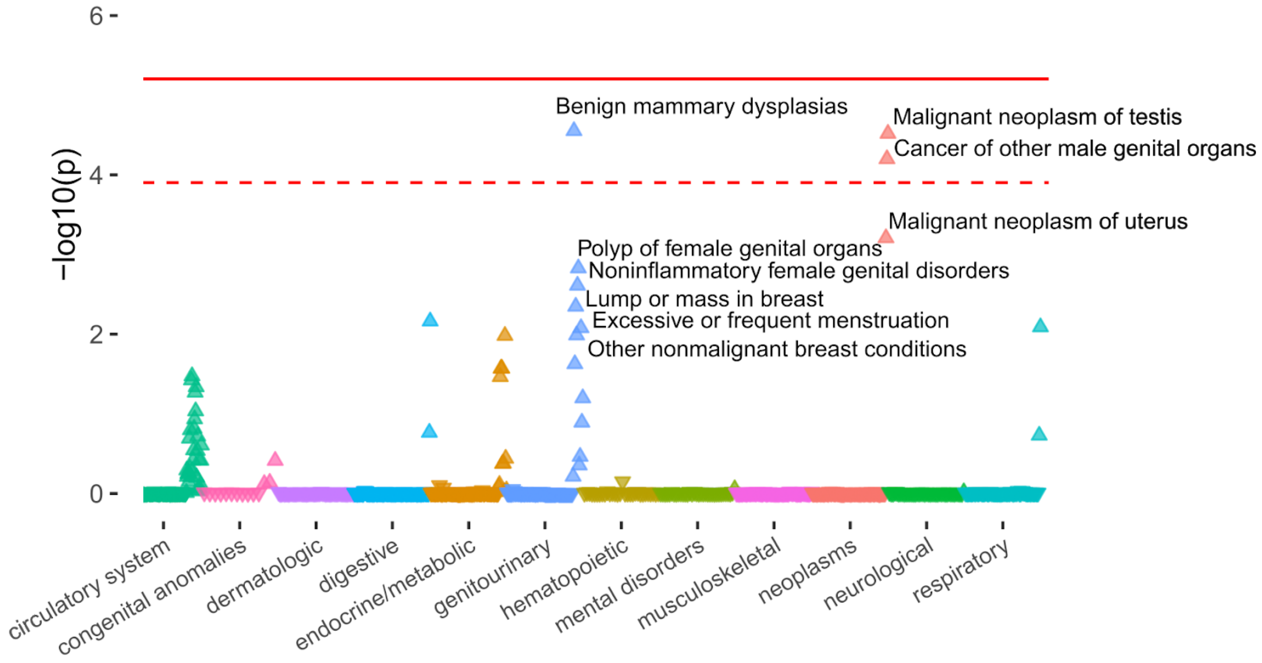

Supplementary Figure 8: PheWAS plot of *BCL2L13* stop-strengthening variant. The solid red line represents the threshold for Bonferroni-adjusted significance (P=6.25e-6) and the red dashed line represents the FDR threshold (P=1.25e-4) adjusted for multiple hypothesis testing. The direction of each arrowhead corresponds to increased risk (up) or decreased risk (down). P-values are derived from a logistic regression model. Red solid line indicates Bonferroni significance threshold (P=6.25e-05). Red dashed line represents the FDR < 0.1 threshold.

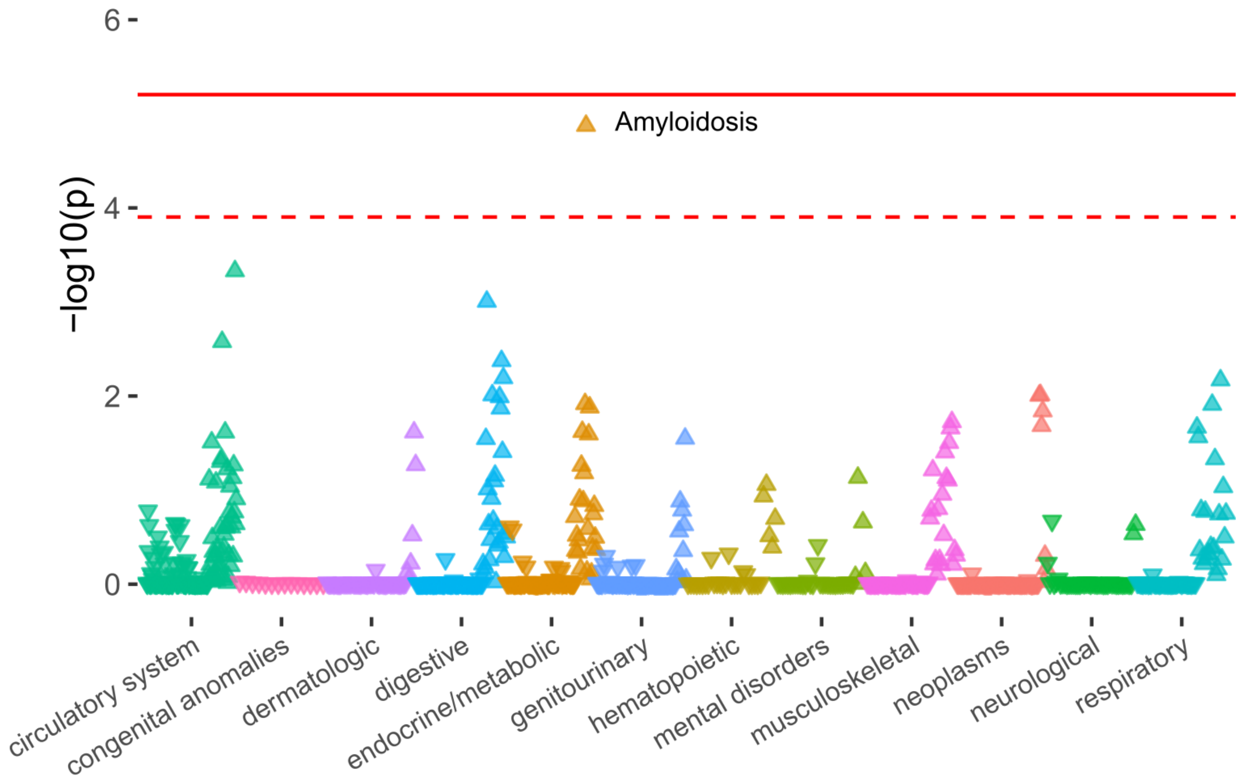

Supplementary Figure 9: PheWAS plot of *NALCN* UAA UTC variant. The solid red line represents the threshold for Bonferroni-adjusted significance (P=6.25e-6) and the red dashed line represents the FDR threshold (P=1.25e-4) adjusted for multiple hypothesis testing. The direction of each arrowhead corresponds to increased risk (up) or decreased risk (down). P-values are derived from a logistic regression model. Red solid line indicates Bonferroni significance threshold (P=6.25e-05). Red dashed line represents the FDR < 0.1 threshold.

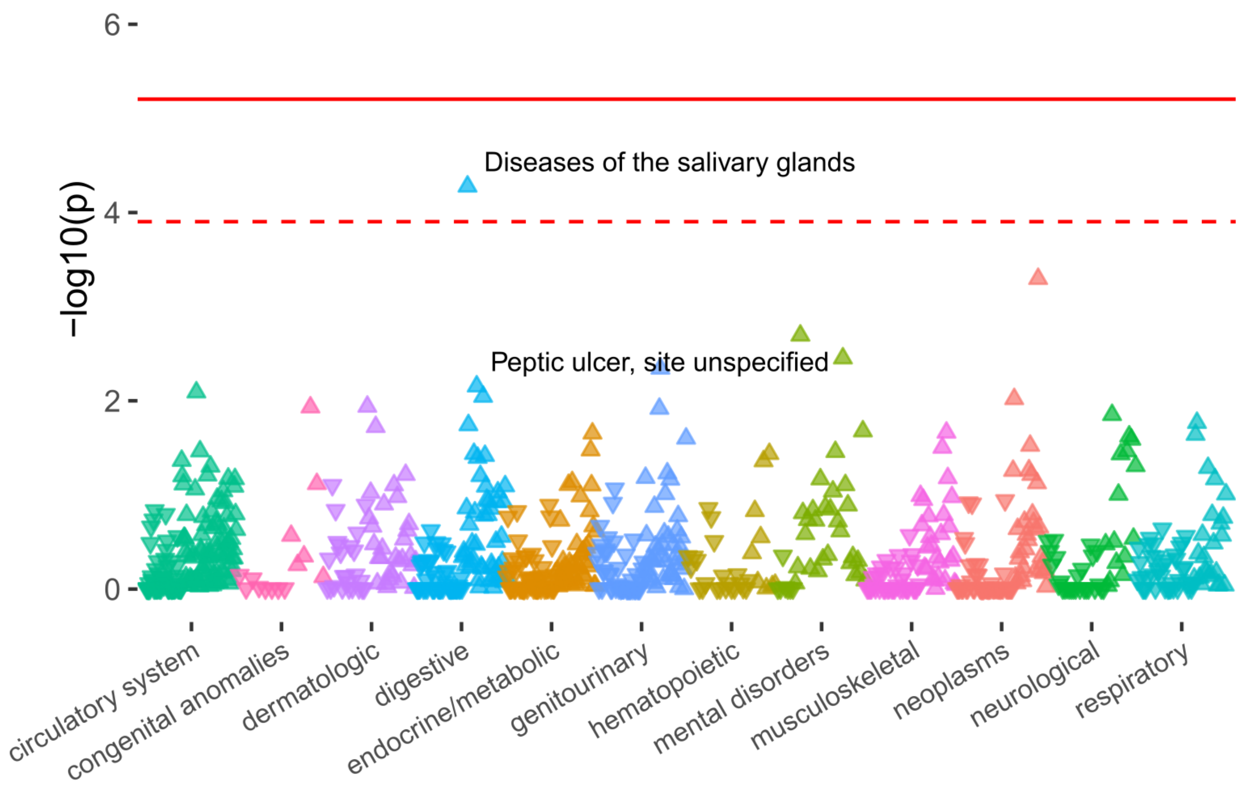

Supplementary Figure 10: PheWAS plot of *SHMT2* stop-strengthening variant. The solid red line represents the threshold for Bonferroni-adjusted significance (P=6.25e-6) and the red dashed line represents the FDR threshold (P=1.25e-4) adjusted for multiple hypothesis testing. The direction of each arrowhead corresponds to increased risk (up) or decreased risk (down). P-values are derived from a logistic regression model. Red solid line indicates Bonferroni significance threshold (P=6.25e-05). Red dashed line represents the FDR < 0.1 threshold.

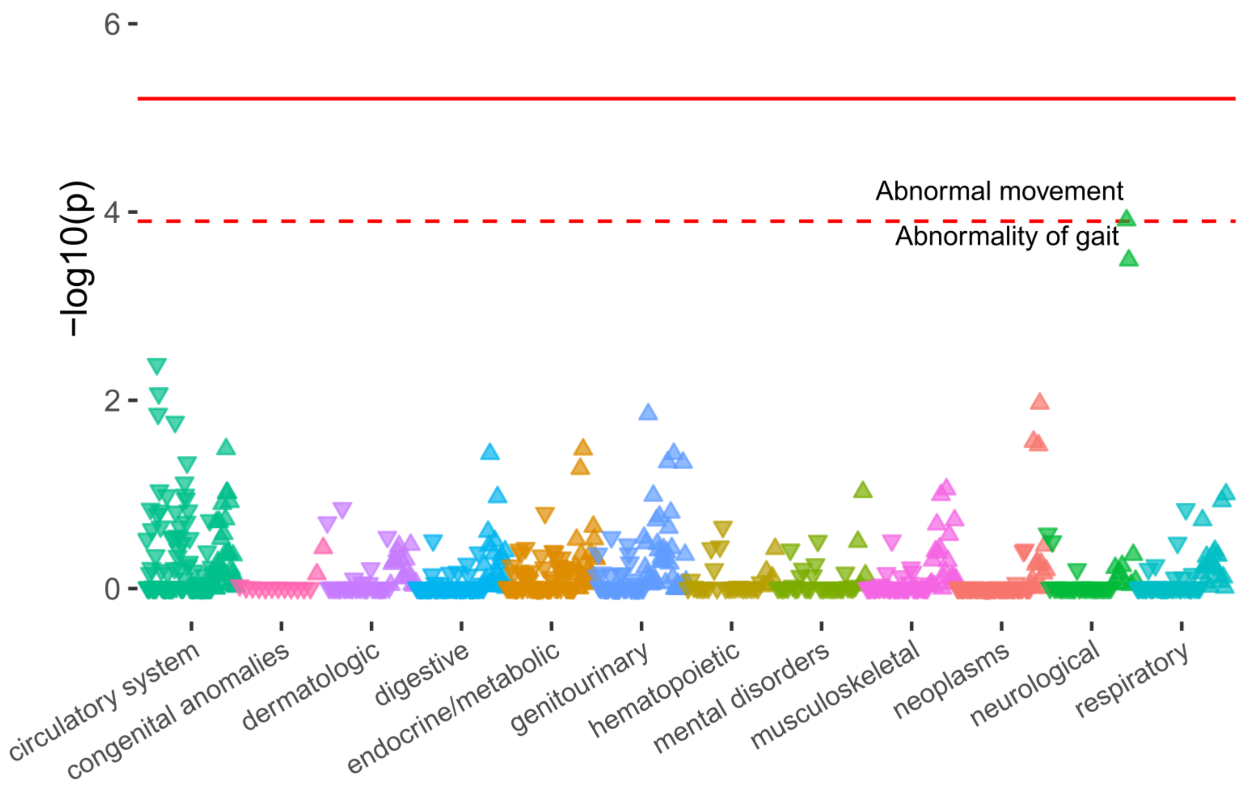

Supplementary Figure 11: PheWAS plot of *MOAP1* UAA UTC variant. The solid red line represents the threshold for Bonferroni-adjusted significance (P=6.25e-6) and the red dashed line represents the FDR threshold (P=1.25e-4) adjusted for multiple hypothesis testing. The direction of each arrowhead corresponds to increased risk (up) or decreased risk (down). P-values are derived from a logistic regression model. Red solid line indicates Bonferroni significance threshold (P=6.25e-05). Red dashed line represents the FDR < 0.1 threshold.

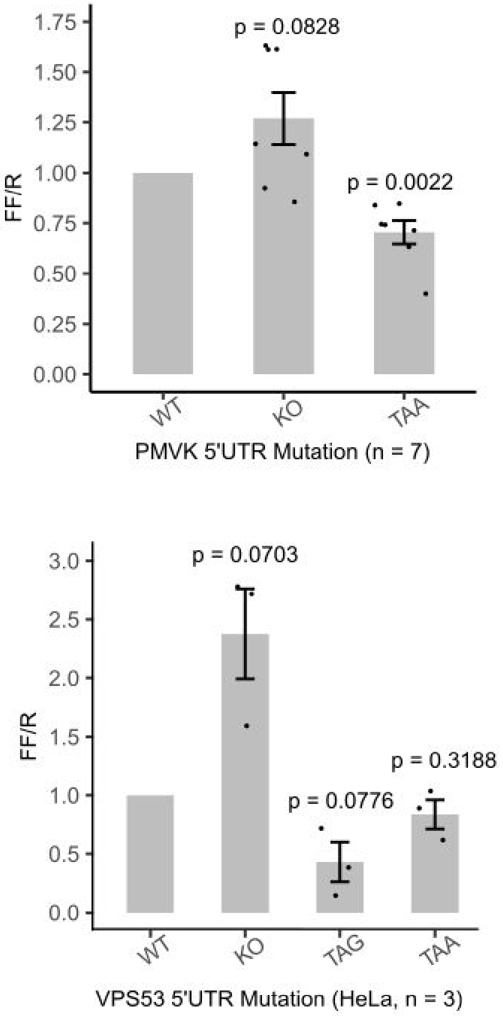

Supplementary Figure 12: Luciferase experiments for *PMVK* and *VPS53* plasmid Constructs showing similar direction of effect for UTC and stop-strengthening variants using HeLa cells for transfection. Results displayed are from independent biological replicates. P-values from one-sample T-test for each condition are displayed above each column. Error bars represent standard error of the mean. Source data are included in the Source Data file.

Supplementary Table 1: uORF UTC / Stop-strengthening MAPS analysis with all CDS-overlapping variants removed.

uORF Variant Type	Number of SNVs	SNVs overlapping any CDS (%)	Original MAPS (95% CI)	MAPS with CDS-overlap removed (95% CI)
UTC	3024	163 (5.39%)	0.0373 (0.0194-0.0552)	0.0362 (0.0172-0.0552)
TAA gained	928	93 (10.02%)	0.0726 (0.0407-0.1045)	0.0765 (0.0430-0.1101)
Stop > TAA	499	51 (10.22%)	0.0971 (0.0537-0.1404)	0.1051 (0.0595-0.1507)

Supplementary Table 2: Relative frequencies of TGA, TAG, and UAA trinucleotides across different 5'UTR sequence contexts

Trinucleotide	Context	Proportion	2.5% quantile	97.5% quantile
UAA	All 5'UTRs	0.354813	0.347512	0.362177
UAA	5'UTRs w/ uORF	0.375554	0.355256	0.396686
UAA	uORF	0.196608	0.17912	0.214626
UAG	All 5'UTRs	0.227442	0.221035	0.233975
UAG	5'UTRs w/ uORF	0.229501	0.211289	0.248058
UAG	uORF	0.285639	0.265501	0.305776
UGA	All 5'UTRs	0.417745	0.410151	0.425083
UGA	5'UTRs w/ uORF	0.394945	0.3739	0.415847
UGA	uORF	0.517753	0.495495	0.540541

Supplementary Table 3: Minor allele frequencies for all PheWAS-significant variants tested in discovery and replication analyses

Gene	Carreir Freq (PMBB)	PMBB EUR	PMBB AFR	Carrier Freq (UKB)	LOF Allele Freq. (PMBB)	LOF Allele Freq. (UKB)
PMVK	0.00298	0.00336	0.00000	0.00324	0.00086	0.00094
VPS53	0.09468	0.10954	0.01940	0.14370	0.00131	0.00109
NALCN	0.00147	0.00129	0.00106	0.00084	0.00122	0.00162
BCL2L13	0.00028	0.00039	0.00190	0.00029	0.00028	0.00025
SHMT2	0.01266	0.00540	0.03715	0.00749	0.00182	0.00033
MOAP1	0.00188	0.00006	0.00793	N/A	0.00026	N/A

Supplementary Table 4: PheWAS replication analyses phenotypes tested

							Single Variant		pLoF Gene Burden			
Variant	Gene	PheCode	Cases (UKB)	Controls (UKB)	Cases (PMBB)	Controls (PMBB)	OR (UKB)	Rep. P (UKB)	OR (UKB)	Rep. P (UKB)	OR PMBB	Rep. P (PMBB)
rs181302437	*PMVK*	250.13	33	32689	23	5198	3.3E-06	0.986	15.82	0.0073	2.46E-05	0.9887
rs181302437	*PMVK*	250.14	13	32689	25	5198	N/A	N/A	N/A	N/A	2.48E-05	0.989
rs181302437	*PMVK*	250.22	11	32689	315	6134	N/A	N/A	N/A	N/A	5.96E-05	0.9672
rs35915949	*VPS53*	300.1	627	31247	1060	6939	0.88	0.1631	1.35	0.6751	1	0.8052
rs35915949	*VPS53*	300	684	31247	1249	6939	0.88	0.1757	1.48	0.5833	1	0.5808
rs139848407	*NALCN*	270.33	11	34565	30	7727	N/A	N/A	N/A	N/A	7.25E-06	0.9892
rs139848407	*NALCN*	270	34	34554	134	9594	9.35E-06	0.9887	9.74	0.0264	4.42	0.1518
rs140799351	*BCL2L13*	610	277	33848	55	7689	1.77E-04	0.973	N/A	N/A	N/A	N/A
rs140799351	*BCL2L13*	187	51	33957	26	7700	56.02	0.0003	N/A	N/A	N/A	N/A
rs140799351	*BCL2L13*	187.2	30	33957	34	7700	79.78	0.0002	N/A	N/A	N/A	N/A
rs28365863	*SHMT2*	527	N/A	N/A	90	9774	N/A	N/A	N/A	N/A	2	0.0055
rs116450723	*MOAP1*	350	N/A	N/A	362	9415	N/A	N/A	N/A	N/A	2.35E-05	0.9659

Supplementary Table 5: 5'UTR Fragments used in expression constructs

Gene (Transcript)	Mutation	Sequence
PMVK (ENST00000368467)	WT	TATAGGGAGACCCAAGCTGGCTAGTTAAGCTTAGATCTTGA<br>TATCCTCGAGAGAAGGTTCTGGGCGGGGCTGGACTGTTCTA<br>AGTGAGTTCGGGTGGGGGAGCTTCACGAGGGGAGGCTGCT<br>CTGTGAAGGAACCGCCTTTCTCTCCGCGTGTCTCACCCTTT<br>TCTCCCCATATCTGTTTGGACATGAGCTGAGGGCACGGTCG<br>CGGGCGGTCAGCCCTGTTCGCAGCTACGGCGAGGAGGGG<br>CGCGATTGTTCCTTGTTGCCGCTCCGCTTAGTGGCCGCGTC<br>CATTCCGCGCGGTGTCCCGATTTTAGGGGTAGGGAGAAGT<br>GTCAGCTTCAGGCATCGCGAGGCGTGGCGGCCCCATGGAA<br>GATGCCAAAAACATTAAGAAGGGCCCAGCGCCATTCTACCC<br>ACTCGAAGACGGGACCGCCGGCGAGCAGCTGCACAAAGCC<br>ATGA
PMVK (ENST00000368467)	KO	TATAGGGAGACCCAAGCTGGCTAGTTAAGCTTAGATCTTGA<br>TATCCTCGAGAGAAGGTTCTGGGCGGGGCTGGACTGTTCTA<br>AGTGAGTTCGGGTGGGGGAGCTTCACGAGGGGAGGCTGCT<br>CTGTGAAGGAACCGCCTTTCTCTCCGCGTGTCTCACCCTTT<br>TCTCCCCATATCTGTTTGGACATGAGCTGAGGGCACGGTCG<br>CGGGCGGTCAGCCCTGTTCGCAGCTACGGCGAGGAGGGG<br>CGCGATTGTTCCTTGTTGCCGCTCCGCTTAGTGGCCGCGTC<br>CATTCCGCGCGTTTTCCCGATTTTAGGGGTAGGGAGAAGTG<br>TCAGCTTCAGGCATCGCGAGGCGTGGCGGCCCCATGGAAG<br>ATGCCAAAAACATTAAGAAGGGCCCAGCGCCATTCTACCCA<br>CTCGAAGACGGGACCGCCGGCGAGCAGCTGCACAAAGCCA<br>TGA
PMVK (ENST00000368467)	TAG>TAA	TATAGGGAGACCCAAGCTGGCTAGTTAAGCTTAGATCTTGA<br>TATCCTCGAGAGAAGGTTCTGGGCGGGGCTGGACTGTTCTA<br>AGTGAGTTCGGGTGGGGGAGCTTCACGAGGGGAGGCTGCT<br>CTGTGAAGGAACCGCCTTTCTCTCCGCGTGTCTCACCCTTT<br>TCTCCCCATATCTGTTTGGACATGAGCTGAGGGCACGGTCG<br>CGGGCGGTCAGCCCTGTTCGCAGCTACGGCGAGGAGGGG<br>CGCGATTGTTCCTTGTTGCCGCTCCGCTTAGTGGCCGCGTC<br>CATTCCGCGCGGTGTCCCGATTTTAAGGGTAGGGAGAAGT<br>GTCAGCTTCAGGCATCGCGAGGCGTGGCGGCCCCATGGAA<br>GATGCCAAAAACATTAAGAAGGGCCCAGCGCCATTCTACCC<br>ACTCGAAGACGGGACCGCCGGCGAGCAGCTGCACAAAGCC<br>ATGA
VPS53 (ENST00000437048)	WT	TATAGGGAGACCCAAGCTGGCTAGTTAAGCTTAGATCTTGA<br>TATCCTCGAGACTGGGGCCTGGGTGGCGGCTGGAGGCCTG<br>AGTTGGGCTCGCGGCGGGGGTCGGCAGGGGGCCGGGTGG<br>CGGAATGGAAGATGCCAAAAACATTAAGAAGGGCCCAGCG<br>CCATTCTACCCACTCGAAGACGGGACCGCCGGCGAGCAGC<br>TGCACAAAGCCATGA

VPS53 (ENST00000437048)	KO	TATAGGGAGACCCAAGCTGGCTAGTTAAGCTTAGATCTTGA<br>TATCCTCGAGACAGGGGCCTGGGTGGCGGCTGGAGGCCTG<br>AGTTGGGCTCGCGGCGGGGGTCGGCAGGGGGCCGGGTGG<br>CGGAATGGAAGATGCCAAAAACATTAAGAAGGGCCCAGCG<br>CCATTCTACCCACTCGAAGACGGGACCGCCGGCGAGCAGC<br>TGCACAAAGCCATGA
VPS53 (ENST00000437048)	TGA>TAA	TATAGGGAGACCCAAGCTGGCTAGTTAAGCTTAGATCTTGA<br>TATCCTCGAGACTGGGGCCTGGGTGGCGGCTGGAGGCCTA<br>AGTTGGGCTCGCGGCGGGGGTCGGCAGGGGGCCGGGTGG<br>CGGAATGGAAGATGCCAAAAACATTAAGAAGGGCCCAGCG<br>CCATTCTACCCACTCGAAGACGGGACCGCCGGCGAGCAGC<br>TGCACAAAGCCATGA
VPS53 (ENST00000437048)	TGG>TAG	TATAGGGAGACCCAAGCTGGCTAGTTAAGCTTAGATCTTGA<br>TATCCTCGAGACTGGGGCCTGGGTAGCGGCTGGAGGCCTG<br>AGTTGGGCTCGCGGCGGGGGTCGGCAGGGGGCCGGGTGG<br>CGGAATGGAAGATGCCAAAAACATTAAGAAGGGCCCAGCG<br>CCATTCTACCCACTCGAAGACGGGACCGCCGGCGAGCAGC<br>TGCACAAAGCCATGA
BCL2L13 (ENST00000543133)	WT	TCGGAGCACTCACCGCCGCTGGGGGACCCTGTCGGAAGCA<br>ACTGCCGCCGCCGCCTCTTTCATCTCTTCTGGGGCAGGGG<br>CCAGGGCCAGGTTTTACACATCCATAAGTAGACCTTTTTGG<br>AGCCTCACCAGCCAATTCAATGGCGTCCTCTTCTACTGTGC<br>CT<br>CTGGGATTTCACTATGAAACAAAGTATGTTGTTCTCAGCTAC<br>TTGGGACTCCTCTCTCAAGAGAAGCTGCAAGAGCAACATCT<br>TTCCTCACCCCAAGGGGTTCAACTAGATATAGCTTCACAATC<br>TCTGGATCAAGAAATTTTATTAAAAGTTAAAACTGAAATTGAA<br>GAAGAGCTAAAATCTCTGGACAAAGAAATTTCTGAAGGCCA<br>GTGACATATCAGGCATTTCGGGAATGTACACTGGAGACCAC<br>AGTTCATGCCAGCGGCTGGAATAAGATTTTGGTGCCTCTGG<br>TTTTGCTACGACAA
BCL2L13 (ENST00000543133)	ATG>ATA	TCGGAGCACTCACCGCCGCTGGGGGACCCTGTCGGAAGCA<br>ACTGCCGCCGCCGCCTCTTTCATCTCTTCTGGGGCAGGGG<br>CCAGGGCCAGGTTTTACACATCCATAAGTAGACCTTTTTGG<br>AGCCTCACCAGCCAATTCAATAGCGTCCTCTTCTACTGTGC<br>CT<br>CTGGGATTTCACTATGAAACAAAGTATGTTGTTCTCAGCTAC<br>TTGGGACTCCTCTCTCAAGAGAAGCTGCAAGAGCAACATCT<br>TTCCTCACCCCAAGGGGTTCAACTAGATATAGCTTCACAATC<br>TCTGGATCAAGAAATTTTATTAAAAGTTAAAACTGAAATTGAA<br>GAAGAGCTAAAATCTCTGGACAAAGAAATTTCTGAAGGCCA<br>GTGACATATCAGGCATTTCGGGAATGTACACTGGAGACCAC<br>AGTTCATGCCAGCGGCTGGAATAAGATTTTGGTGCCTCTGG<br>TTTTGCTACGACAA
BCL2L13	TGA>TAA	TCGGAGCACTCACCGCCGCTGGGGGACCCTGTCGGAAGCA

(ENST00000543133)		ACTGCCGCCGCCGCCTCTTTCATCTCTTCTGGGGCAGGGG CCAGGGCCAGGTTTTACACATCCATAAGTAGACCTTTTTGG AGCCTCACCAGCCAATTCAATGGCGTCCTCTTCTACTGTGC CT CTGGGATTTCACTATGAAACAAAGTATGTTGTTCTCAGCTAC TTGGGACTCCTCTCTCAAGAGAAGCTGCAAGAGCAACATCT TTCCTCACCCCAAGGGGTTCAACTAGATATAGCTTCACAATC TCTGGATCAAGAAATTTTATTAAAAGTTAAAACTGAAATTGAA GAAGAGCTAAAATCTCTGGACAAAGAAATTTCTGAAGGCCA GTAACATATCAGGCATTTCGGGAATGTACACTGGAGACCAC AGTTCATGCCAGCGGCTGGAATAAGATTTTGGTGCCTCTGG TTTTGCTACGACAA

Supplementary Table 6: Nominal cardiac and movement disorder associations with SHMT2 stop-strengthening variant uncovered through PheWAS in Penn Medicine Biobank.

phenotype	beta	OR	SE	p	n_total	n_cases	n_controls	allele_freq	description
747.13	1.518	4.561	0.602	0.0117	7300	62	7238	0.005410959	Congenital anomalies of great vessels
350.1	1.124	3.077	0.497	0.0238	9519	104	9415	0.011503309	Abnormal involuntary movements
350.2	0.660	1.935	0.296	0.0258	9626	211	9415	0.011790983	Abnormality of gait
426.22	1.635	5.129	0.773	0.0343	2481	50	2431	0.005441354	Mobitz II AV block
972.1	1.434	4.197	0.703	0.0412	6285	40	6245	0.005966587	Cardiac rhythm regulators causing adverse effects in therapeutic use
427.5	0.859	2.362	0.438	0.0498	3412	157	3255	0.013335287	Arrhythmia (cardiac) NOS

Supplementary Data 1: Spreadsheet detailing all potential uORF stop creating positions identified in this analysis.

Supplementary Data 1 is provided as a text file accessible at https://doi.org/10.5281/zenodo.4536050 [71]
