# Supplementary material for: Disrupting upstream translation in mRNAs is associated with human disease
Source: Nat Commun. 2021 Mar 9;12:1515. doi: 10.1038/s41467-021-21812-1 (PMC7943595; doi:10.1038/s41467-021-21812-1)
Supplement: Supplementary file 3 — Reporting Summary [file 41467_2021_21812_MOESM3_ESM.pdf]

nature research

Corresponding author(s): Yoseph Barash, Louis Ghanem

Last updated by author(s): Jan 13, 2021

Reporting Summary

Nature Research wishes to improve the reproducibility of the work that we publish. This form provides structure for consistency and transparency in reporting. For further information on Nature Research policies, see our Editorial Policies and the Editorial Policy Checklist.

Statistics

For all statistical analyses, confirm that the following items are present in the figure legend, table legend, main text, or Methods section.

n/a	Confirmed	
☐	☒	The exact sample size (*n*) for each experimental group/condition, given as a discrete number and unit of measurement
☐	☒	A statement on whether measurements were taken from distinct samples or whether the same sample was measured repeatedly
☐	☒	The statistical test(s) used AND whether they are one- or two-sided<br>*Only common tests should be described solely by name; describe more complex techniques in the Methods section.*
☐	☒	A description of all covariates tested
☐	☒	A description of any assumptions or corrections, such as tests of normality and adjustment for multiple comparisons
☐	☒	A full description of the statistical parameters including central tendency (e.g. means) or other basic estimates (e.g. regression coefficient) AND variation (e.g. standard deviation) or associated estimates of uncertainty (e.g. confidence intervals)
☐	☒	For null hypothesis testing, the test statistic (e.g. *F*, *t*, *r*) with confidence intervals, effect sizes, degrees of freedom and *P* value noted<br>*Give P values as exact values whenever suitable.*
☒	☐	For Bayesian analysis, information on the choice of priors and Markov chain Monte Carlo settings
☐	☒	For hierarchical and complex designs, identification of the appropriate level for tests and full reporting of outcomes
☐	☒	Estimates of effect sizes (e.g. Cohen's *d*, Pearson's *r*), indicating how they were calculated

Our web collection on statistics for biologists contains articles on many of the points above.

Software and code

Policy information about availability of computer code

Data collection	gnomAD whole genomes version 3 release data were downloaded directly from the gnomAD website. PhyloP scores for the conservation analysis were downloaded from UCSC.
Data analysis	Data analysis was completed using R 3.6.1, R 3.3.1, and Python 3.7.3. Code that can be used to reproduce the analyses presented in the paper is available from https://bitbucket.org/biociphers/uorf-paper-2020/src/master/. Additional software used for this paper include ANNOVAR (version 2018Apr16), bedtools (2.27.1), bcftools (1.9), and the Variant Effect Predictor (Ensembl) version 98.2.

For manuscripts utilizing custom algorithms or software that are central to the research but not yet described in published literature, software must be made available to editors and reviewers. We strongly encourage code deposition in a community repository (e.g. GitHub). See the Nature Research guidelines for submitting code & software for further information.

Data

Policy information about availability of data

All manuscripts must include a data availability statement. This statement should provide the following information, where applicable:

- Accession codes, unique identifiers, or web links for publicly available datasets
- A list of figures that have associated raw data
- A description of any restrictions on data availability

The set of gnomAD variants obtained from 71,702 whole genome sequences used for the MAPS analysis are available from https://gnomad.broadinstitute.org/downloads. The set of mapped non-canonical ORFs from ribosome profiling studies used for the analyses presented are available from https://doi.org/10.7554/eLife.08890.023. This set includes 5'UTR (uORF), 3'UTR (dORF), long-noncoding RNA, and pseudogene ORFs mapped by the RibORF algorithm. Raw data from the associated ribosome profiling experiment is deposited in the GEO under accession GSE65885. Codon stability coefficient (CSC) scores used in the analyses were downloaded from https://doi.org/10.7554/eLife.45396.006. Source data are provided with this paper. Individual-level data from the Penn Medicine BioBank are not

publicly available due to research participant privacy concerns; however, requests from accredited researchers for access to individual-level data relevant to this study can be made by contacting the corresponding author. Up-to-date summary data for genetic variants cpatured using WES in the PMBB can be accessed via the PMBB Genome Browser (https://pmbb.med.upenn.edu/allele-frequency/). Base-level conservation phyloP values were obtained from the UCSC Genome Browser at the following link: https://genome.ucsc.edu/cgi-bin/hgTrackUi?db=hg38&g=cons100way.

Field-specific reporting

Please select the one below that is the best fit for your research. If you are not sure, read the appropriate sections before making your selection.

- [x] Life sciences
- [ ] Behavioural & social sciences
- [ ] Ecological, evolutionary & environmental sciences

For a reference copy of the document with all sections, see nature.com/documents/nr-reporting-summary-flat.pdf

Life sciences study design

All studies must disclose on these points even when the disclosure is negative.

Sample size

The discovery analysis in the Penn Medicine Biobank (PMBB) included a subset of 10,900 individuals who have undergone whole-exome sequencing following quality control measures. Only 5' UTR variants with at least five total alternate alleles in PMBB were selected for univariate PheWAS analyses in the discovery phase while variants with greater than half of the genotypes annotated as missing due to low quality were excluded. Additionally we chose to interrogate gene burdens with at least 25 carriers. This resulted in a final set of N=10 variants. Our association analyses considered only disease phenotypes with at least 20 cases, leading to the interrogation of 800 total Phecodes in the PMBB. For discovery and replication analyses in the PMBB (N = 11,451) and UKB (N=32,268), the sample sizes were opportunistic and determined by the availability in each biobank at the time of genetic sequencing.

Data exclusions

In the discovery analysis, out of 11,451 total individuals with whole-exome sequencing in the PMBB, samples with low exome sequencing coverage (less tahn 75% of targeted bases achieving 20x coverage), high missingness (greater than 5% of targeted bases), high heterozygosity, dissimilar reported and genetically determined sex, genetic evidence of sample duplication, and cryptic relatedness, were removed. This resulted in a total of 10,900 individuals following pre-established protocols ofr quility control of exome sequencing data. For phenotypes examined, ICD codes associated with injury and poisonings were excluded under the assumption that htese diagnoses would be less likely to be associated with genetic variation. Additionally patients were determined to have a phenotype label only if they had the corresponding ICD diagnosis on two or more dates. Phenotypic controls consisted of individuals who never had the ICD code. Individuals with an ICD diagnosis on only one date, as well as individuals under control exclusion criteria based on PheWAS phenotype mapping protocols were not considered for statistical analyses. These pre-established phenotypic exclusion criteria were implemented to increase sensitivity for defining cases for disease phenotypes. For replication analyses in the UKB, similar exclusion criteria were implemented with both genotype and phenotype aspects.

Replication

For each significant single-variant and phenotype association uncovered in the analysis of the PMBB, replication was attempted by an independent association study in the UKB and by gene-burden studies in both the PMBB and UKB. Out of 6 variant-phenotype associations uncovered in the discovery analysis, 1 was replicated as a single-variant association in the UKB, and 3 were replicated by loss-of-function gene burden studies in either the UKB or PMBB. Several single-variant associations could not be replicated in the UKB due to insufficient case numbers, or through gene-burden analyses if not enough loss-of-function variants could be identified across genes in the PMBB or UKB respectively.

For replication studies in UKB, we interrogated the 32,268 individuals of European ancestry (based on UKB's reported genetic ancestry grouping) with ICD-10 diagnosis codes available among the 49,960 individuals who had WES data as generated by the Functional Equivalence (FE) pipeline. We focused our replication efforts on 32,268 individuals after removing samples with poor genotype quality, individuals closer than 3rd degree relatives, and those with dissimilar reported and genetically determined sex. The PLINK files for exome sequencing provided by UKB were based on mappings to GRCh38. Access to the UK Biobank for this project was from Application 32133.

Randomization

Each disease phenotype was tested for association with each uORF variant using a logistic regression model adjusted for age, age2, sex, and the first ten principal components (PCs) of genetic ancestry.

Blinding

No blinding was conducted. The data analyzed are from large population cohorts collected independently by the Penn Medicine Biobank and UK Biobank teams who had no prior knowledge of the planned analyses.

Reporting for specific materials, systems and methods

We require information from authors about some types of materials, experimental systems and methods used in many studies. Here, indicate whether each material, system or method listed is relevant to your study. If you are not sure if a list item applies to your research, read the appropriate section before selecting a response.

Materials & experimental systems

n/a	Involved in the study
☒	☐ Antibodies
☐	☒ Eukaryotic cell lines
☒	☐ Palaeontology and archaeology
☒	☐ Animals and other organisms
☐	☒ Human research participants
☒	☐ Clinical data
☒	☐ Dual use research of concern

Methods

n/a	Involved in the study
☒	☐ ChIP-seq
☒	☐ Flow cytometry
☒	☐ MRI-based neuroimaging

Eukaryotic cell lines

Policy information about cell lines

Cell line source(s)	HeLa cells were obtained as a gift from the Adny Minn Lab at the University of Pennsylvania (originally purchased from ATCC). HEK293T cells were obtained as a gift form the Yana Kamberov Lab at the University of Pennsylvania (originally purchased from ATCC).
Authentication	Cell lines were not authenticated.
Mycoplasma contamination	Cell lines were not tested for mycoplasma contamination.
Commonly misidentified lines (See ICLAC register)	Commonly misidentified cell lines were not used.

Human research participants

Policy information about studies involving human research participants

Population characteristics	A subset of 10,900 individuals in PMBB were analyzed in the discovery phase of this study having a median age of 67 with the two most prevalent ancestries being Europeans (75.2%) and Africans (19.9%). 41% of the participants in the PMBB data used for this study are female. The UK Biobank cohort consisted of 32,268 individuals of European ancestry with a median age of 59. 55% of the participants in the UKB data used for this study are female.
Recruitment	All individuals who were recruited for the Penn Medicine Biobank (PMBB) are patients of clinical practice sites of the University of Pennsylvania Health System. Appropriate consent was obtained from each participant regarding storage of biological specimens, genetic sequencing, access to all available electronic health record (EHR) data, and permission to recontact for future studies.<br><br>Being a hospital-based biobank drawing from the patient population in the University of Pennsylvania hospital system, it is possible that recruitment and participation in the study population is affected by self-selection bias due to a combination of geographic, historical, and socioeconomic factors that are not explicitly addressed in the present study design. Where possible we have attempted to replicate gene-phenotype associations in a separate biobank drawing from a different population, however it remains unclear how these additional factors might impact the study results in our discovery analysis.
Ethics oversight	The study was approved by the Institutional Review Board of the University of Pennsylvania and complied with the principles set out in the Declaration of Helsinki. Replication analyses were conducted using the whole exome sequencing (WES) dataset from the UK Biobank (UKB).

Note that full information on the approval of the study protocol must also be provided in the manuscript.
